# The relationship of emotional abuse, self-value and conflict resolution needs in secondary school students

**DOI:** 10.3389/fpsyg.2022.966702

**Published:** 2022-11-24

**Authors:** Tümen Erses, Yalin Kiliç, Bengü Berkmen

**Affiliations:** ^1^Department of Guidance and Psychological Counseling, Faculty of Education, Cyprus International University, Nicosia, Cyprus; ^2^Department of Psychology, Faculty of Arts and Sciences, Cyprus International University, Nicosia, Cyprus

**Keywords:** conflict resolution, teenager, emotional exploitation, ecological model, social anxiety, self-respect

## Abstract

The most crucial period for the teenagers is the secondary school period. The affection and knowledge that lead the student to build up his or her own self individuality, develop positive relationships, and cope with possible conflicts must be given to her or him by his or her family and neighborhood. The aim of this study was to analyze the correlation among the self-respect, the need for the solution of the conflict, social anxiety, and emotional exploitation within the range of the middle school students. The aforementioned research is such a kind of descriptive study which used one of the correlational survey research models, the quantitative method. In addition to the Personal Data Collection Document, the Scales of the Need of Conflict Resolution, emotional exploitation, the Rosenberg Self-Respect Scale, and the Social Anxiety Scale for the Teenagers are used. The research universe of this study is composed of 10.196 middle school students studying in the 6^th^, 7^th^, and the 8^th^ year, at the public schools of the Ministry of Education of the TRNC. The sample group of the research is constituted by 530 volunteer middle school students from Famagusta, Guzelyurt, Lefke, Nicosia, and Kyrenia, respectively. The AMOS 2.1 and the SPSS 25 are used to resolve the data collected. The study that carried out through the structural equality method verifies that there is an agent role of the Need for the Conflict Resolution in between the self-respect and emotional exploitation. However, there cannot be verified any relation in between the self-respect and the social anxiety within the agent role of the Need for the Conflict Resolution. There has been verified meaningful and positive correlations among through the marks of the students taken from the NCR and the SAST, the NCR and the EES, the NCR and the RSRS, the SAST and the RSRS, the SAST and the EES, and the EES and the RSRS.

## Introduction

Conflict is a situation of discussion, tension, and disagreement among one person or more on any subject (Öner, 2004). It is known that how adolescents experiencing conflict impacted psychologically and physically is not a one-dimensional problem arising from their own characteristics, but a multidimensional problem that is also affected by school, family, teachers, friends, and culture (Dogan, 2010). Conflict resolution is the ability of the proponent to come together to solve the problem (Koruklu et al., 2016).

Adolescence means that the student changes in many ways in terms of physiological, psychological, cognitive, and sociological aspects. Adolescents grow, mature, develop, change, and try to cope with personal, academic, and social problems. To pass this period in a healthy way, the adolescent needs to acquire problem-solving skills. Problem solving is how the adolescent will behave to overcome the obstacles he encounters to reach his goal. It is seen that intelligence is important in problem-solving skills, which can also be revealed through learning. The most important factors in the development of these abilities are their families and close circles (Arslan and Kabasakal, 2013).

Emotional abuse is all the types of behavior that psychologically harm the person and is mostly experienced during adolescence (Kanak and Çelik, 2018). In emotional abuse, children may lose their self-confidence, be hurt, and experience a decrease in respect (Curun, 2021). Emotionally abused adolescents will experience aggressive, negative self-development, disorder in social relations, and the lack of self-confidence (Yalçin, 2007). Abuse is the deprivation of children of attention and love (Koçmarlar and Akbag, 2018). Self-concept is to perceive what the student is, what he/she wants to do, and why (Biyikli, 2014). It occurs as a result of social interactions and reflects the student's self-awareness. It is the true personality of individuals. The self-perception of the student is his/her selfhood (Özyürek et al., 2020). Social anxiety is the avoidance of all social situations such as speaking, being introduced, and eating in a crowded environment. The individual has the feeling that he is constantly being followed by others (Erkan, 2002). Social anxiety, which is mostly experienced at the age of 11–17, reduces the quality of making friends for adolescents and decreases their social interactions and quality of life (Kermen et al., 2016).

Today's rapid developments and changes also bring problems. It is a known fact that in youth, the student experiences conflicts and problems with her/his family and environment. In the study, which was carried out to measure what can be done against the conflicts experienced by young people who can keep up with technological developments, it was emphasized that these problems can be prevented by applying conflict resolution education and communication skills, in which students have conflicts with their families and surroundings in this period, and there can be peace, happiness, and success in the society (Dinçyürek and Civelek, 2008). Adolescents are afraid of being misunderstood and being alone. In adolescence, students may be afraid of being alone because they have difficulty in communicating with parents and social media. It is important that the problem-solving skills of the person are at a high level, so that the student is prepared for the future in the best way (Armagan and Eşkisu, 2011). Students who need to use their problem-solving skills will be able to be successful in their schools and lives without having conflicts by generating solutions to the problems they encounter (Ekici and Balim, 2013).

The first relationship established with the mother and father leads to his selfness and to close relations he will establish with his friends, teachers, and environment. In other words, negative parental attitudes, which means not meeting the needs of the individual on a sound basis, such as criticizing, rejecting, overprotecting, and neglecting, can lead to the development of lovelessness, worthlessness, and negative beliefs. It has been observed that as a result of this situation, which is perceived in childhood, a tendency for individuals to experience conflict in their interpersonal relationships during adolescence (Cesur et al., 2017). Family, teachers, and friends have a very important role in the lives of adolescents. As the adolescent's social support from friends, teachers, and family increases, their conflict resolution skills also increase, and as social support from friends increases, their social development will increase and they will be able to establish better relationships (Karapinar, 2021). Since young people who can establish positive relationships will have the motivation and happiness to fulfill their expectations, the satisfaction they will get from their life will increase in accordance with this situation (Akyürek et al., 2018). Adolescents who are criticized by their parents and have a deficiency of love develop passive, diffident, and antisocial behaviors (Öztep, 2010). Behaviors of parents that lead to emotional abuse cause both emotional and behavioral problems in students (Ulu, 2011).

It has been determined that the adolescents who experience conflict are those with low self-esteem, poor social adaptation, communication problems, and spend most of their time alone (Akyol and Bilbay, 2018). Meaningful negative and positive relationships were found among conflict resolution, aggression, and anger (Gündogdu, 2010). Event scales were administered to 210 Chinese university students and it was observed that socially weak students who do not have close friends or who lost them are nervous and quiet with unfamiliar people. It was found that negative life events mediated fully in social avoidance (Li et al., 2013). School is a place where both students' cognitive, physical, and socio-emotional development continues and social interactions, different cultures, and perspectives are brought together. School years in which their selfness develops are the ages that present positive and negative experiences for students (Öztürk, 2019). The most common situations that cause a conflict in schools can be seen as disrupting the game the students are playing, hurting each other, making fun of each other, using nicknames, and taking their belongings without permission (Koruklu, 2008).

While conflicts and threats in schools cause students to stay away from their schools and fail academically, they should be taught to solve conflict problems rather than engage in conflict. There is a strong relationship between conflict among students and academic failure. With prevention and intervention programs in schools, the focus can be on changing students' personality traits (e.g., beliefs, attitudes, and behaviors) and creating a more moderate school environment (Evoy and Welker, 2000). Conflict is a condition that our children must experience for their social development. It has been observed that if the student's social emotional skills improve, the aggressive behaviors they display will lessen, and they will be constructive in conflict resolution (Koruklu et al., 2017). Developmental and preventive guidance services are increasing more and more to prevent conflicts without any problems beforehand to fight with the increasing violence, conflict events, and disagreements among students in schools and to increase students' conflict resolution skills (Koruklu et al., 2016).

A student who recognizes and is aware of his/her feelings can spare time and energy to get to know and understand other students. The student who does not know herself/ himself and constantly has problems with herself cannot focus on getting to know others. The relationship established with parents and children and the behaviors in between are crucial in the development of self-perception (Yüksel, 2009). Emotion can be used. Students can be grouped by interaction, shared culture, shared group interests. Students who share each other's feelings build their relationships in a positive way. Emotion goes with identity (Mercer, 2014). Self-perception is the value she/he gives to herself/himself and the respect she/he feels. A student who successfully experiences adolescence without any difference between his/her own self and his/her ideal self will contribute to the social order, entrepreneurship, and self-confidence of the student when she/he can get enough of his high self-esteem and sense of approval from his/her friend, school, and parents in this period (Aliyev and Erhan, 2017).

Development is a coherent, life-long, continuous change and progress. The person shows a development according to age, with different characteristics. Middle childhood and early adolescence are important developmental times in which students aged 6–14 form their identities. Biological and cognitive changes change students' bodies and minds. Social relationships and roles change dramatically as students build relationships. In middle childhood, students develop self-esteem and a sense of individuality by comparing themselves to their friends (Eccles, 1999). Experiences with their families and friends during these developmental periods can have lasting effects on the child. First of all, a person's development of a healthy personality in aspects of social and emotional development during preschool is related to adaptation to her/his environment. If parents support their children socially and emotionally, they develop a personality structure that is self-confident, independent, creative, able to protect their own rights, able to cooperate with their environment, love themselves, and love their environment, all of which occurs in balance and harmony (Kandir, 2008). Students' lives are full of decisions they make, and their lives become meaningful with these decisions. To be happy in life, individuals should take their own responsibility at the time they want and under desired conditions they wish, form their own identity, and develop their own hidden powers. Students with high self-esteem have their own power to easily determine what they want in life and prevent the emergence of problems (Terzi, 2016). The person strives to understand her/his inner world and the reason for her/his behavior at every moment. Here, self-esteem is a part of his behavior and guides the person in his inner journey. Self-esteem is the value a person gives to herself/himself, positive or negative thoughts about herself/himself. It is the self-confidence of a person throughout her life in every developmental period (Mert et al., 2019).

Students may experience problems in their behaviors and personalities when they cannot communicate properly and are emotionally abused. Such students may have problems in their relationships with their family, environment, and friends and get to be alone, and in overprotective situations dictated by parents, addiction may develop among students and prevent the development of positive self-concepts. Emotional abuse in childhood is strongly associated with depressive symptoms in students. Emotional abuse in childhood; it is associated with behavior problems, hyperactivity, peer problems, and depressive symptoms (Li et al., 2021). Thus, students cannot maintain healthy communication and may experience conflict with their social circle, friends, and family (Karakuş, 2006). Students with low self-esteem feel unsuccessful, worthless, angry, and in conflict with their family, friends, and social circle. A student with high self-esteem develops a better sense of self-confidence, is successful, creative, assertive, can express her/his ideas, and can establish socially harmonious relations with her/his friends, family, and environment (Sarikaya, 2015). It has been shown that students with low self-esteem experience failure and this causes them to feel ashamed and worthless after failure (Brummelman et al., 2014).

Anxiety disorder starts at an early age and the most dangerous ages are between 10 and 15. Many adolescents have difficulties in their students' life and experience incurable and unrecognized anxiety disorders. In the study conducted using the Anxiety Screen Scale, it is seen that all specific anxiety disorders are experienced significantly (Kirubasankar et al., 2021). Their hands shake, their faces blush, and they are afraid of doing something wrong. It is commonly seen that adolescents with such behaviors receive help (Öztürk and Uluşahin, 2008). Adolescents who do not have a friend or a social circle will also be anxious in social environments because of the fear of being criticized as a result of considering the conversations directed toward them (Eriş and Ikiz, 2013). If the adolescent perceives her/his body negatively and she/he experiences social introversion, the relationship between social relationship and body image is important. Students experience social anxiety at most when they encounter new situations and are negatively evaluated (Aslan and Koç, 2018).

This study aims to examine the relationship between secondary school students' self-esteem, their need for conflict resolution and social anxiety, and psychological abuse. The problem statement of the study was determined as what the scale of the relationship between self-esteem, need of conflict resolution, social anxiety, and emotional abuse are among secondary school students.

### Sub problems

1) Is there a meaningful difference in the prediction level of the students according to the scores they get between the Conflict Resolution Needs Scale, and Rosenberg Self-Esteem Scale?
2) Is there a significant difference in the predictive level of the students' Conflict Resolution Needs Scale scores from the Rosenberg Self-Esteem Scale?
3) Is there a significant difference in the predictive level of the scores of the students' Social Anxiety Scale for Adolescents and the Rosenberg Self-Esteem Scale scores?
4) Is there a significant difference in the predictive level of the Emotional Abuse Scale scores of the students and the scores they got from the Rosenberg Self-Esteem Scale?
5) Do students' Conflict Resolution Needs Scale scores have a mediating role between Adolescent Social Anxiety and Psychological Abuse Scale scores and Rosenberg Self-Esteem Scale scores?

## Methods

### Model of the research

Relational research is a type of correlational research which to examine the relationship among the variables and especially the degree of the relationship between two or more quantitative variables (Sönmez and Alacapinar, 2014). In the study, structural equation modeling (SEM) techniques were used to test the correlational relationships among the variables. It is a multivariate method and is completely based on the theory (Tüfekçi and Tüfekçi, 2006).

### Working group

The population of the study is consisted of a total of 10.196 Ministry of Education, 2021 6th-, 7th-, and 8th-grade secondary school students affiliated to the Ministry of National Education in the K. K. T. C.; with the known sampling formula, 95% confidence level, 5% sampling error, a number of individuals to be interviewed were determined as 384, and with the voluntarily participated in the research to reduce the sampling error; the total count became 530 as 347 girls and 183 boys (Sönmez and Alacapinar, 2014). It is important to calculate the sample size in studies. It is not possible to study the entire population. A set of participants representing the population can be selected and accurate inferences can be made from the results. This set of individuals is called a “sample.” This example is a part that represents a whole (Kadam and Bhalerao, 2010).

Table 1 gives the distribution of the individuals included in the study according to their socio-demographic information.

**Table 1:** Distribution of students according to their socio-demographic characteristics.

		***n***	**%**
Gender	Girl	347	65.47
	Boy	183	34.53
Age	12 y.o.	124	23.40
	13 y.o.	224	42.26
	14 y.o.	182	34.34
Grade	6. grade	75	14.15
	7. grade	213	40.19
	8. grade	242	45.66
Number of siblings	Single	54	10.19
	One	266	50.19
	Two	148	27.92
	Three or more	62	11.70
Parents marital status	Married	437	82.45
	Single/divorced	93	17.55
Mother's education level	Uneducated	32	6.04
	Primary school	122	23.02
	Secondary school	82	15.47
	High school	191	36.04
	Undergraduate	103	19.43
Father's education level	Uneducated	18	3.40
	Primary school	105	19.81
	Secondary school	104	19.62
	High school	204	38.49
	Undergraduate	99	18.68
Mother work level	Working	249	46.98
	Unemployed	281	53.02
Father work level	Working	506	95.47
	Unemployed	24	4.53

According to the data obtained from the table, 65.47% of the students are girls, 34.53% are boys, and 23.40% are 12 years old, 42.26% are 13 years old, and 34.34% are 14 years old. In total, 14.15% of the individuals are 6th grade, 40.19% are 7th grade, and 45.66% are 8th grade.

In the study among the individuals who are participated, 10.19% do not have siblings, 50.19% have one sibling, 27.92% have two siblings, and 11.70% have three or more siblings. Parents of 82.45% of individuals are married, and 17.55% of them are divorced/separated. Considering the educational status of the mothers of the students, it is seen that the mothers of 6.04% did not complete a school, 23.02% completed primary school, 15.47% secondary school, 36.04% high school, and 19.43% undergraduate. Considering the educational status of their fathers, it is seen that 3.40% of them have not completed a school, 19.81% of them have completed primary school, 19.62% of them have completed secondary school, 38.49% of them have completed high school, and 18.68% of them have graduated from undergraduate. The mothers of 46.98% of the students are working, 53.02% of them are not working, and fathers of 95.47% are employed whereas 4.53% of them are unemployed.

### Data collection tools

Conflict Resolution Needs Scale (Koruklu et al., 2016), Emotional Abuse Scale (Arslan, 2015), Rosenberg Self-Esteem Scale (Çuhadaroglu, 1986), Social Anxiety Scale for Adolescents (Aydin and Sütcü, 2007), and Personal Information Form were used to collect data during the study.

#### Conflict Resolution Needs Scale

Adapted by Koruklu et al. (2016), the things measured consist of the followings: Conflict Resolution Skills, Conflict Resolution, Need for Conflict Resolution, and Conflict Resolution Scale One Dimension, 19 items. The scale type is Self-Report. It was graded as a 5-point Likert scale (1 = very difficult, 2 = hard, 3 = medium, 4 = easy, and 5 = very easy) that can be applied to secondary school students, high school students, and adolescents. According to the scoring of the scale, the high scores indicate that the student's needs for developing conflict resolution skills are also high. The internal consistency of item-total correlations was examined, and it was stated that the internal consistency coefficient was 0.91, and the Conflict Resolution Scale could be used on secondary school students to determine the level of the students' needs of conflict resolution skills (Koruklu et al., 2016).

#### Emotional Abuse Scale

Developed by Arslan (2015), the characteristics that are measured and their sub-dimensions are emotional abuse, humiliation-intimidation, refusal to react emotionally, isolation-rejection, and delinquency, which consists of a total of 27 items and four sub-dimensions. The type of the scale, self-report, was rated on a 4-point Likert scale that can be applied to adolescents (1 = never, 2 = rarely, 3 = often, and 4 = always). It is stated that the emotional abuse perceived from the parents is high if they get a high score from the scale individually or over the total score. The internal consistency of item-total correlations was examined and the internal consistency coefficient was 0.93. The sub-dimension internal consistency coefficients were determined between 0.83 and 0.93. It was stated that there was a significant relationship between the scales in which negative self and depression were used within the scope of validity and psychological abuse, and that the scale could be used in the evaluation of emotional abuse in adolescents (Arslan, 2015).

#### Social Anxiety Scale for adolescents

Adapted and developed by Aydin and Sütcü (2007), the characteristics measured and their sub-dimensions are social anxiety in adolescents, social avoidance—feeling uneasy, social avoidance in new places—feeling uneasy, and fear of negative evaluation in general, which totally consists of 18 items and three sub-dimensions. The type of the scale was Self-Report, graded as a 5-point Likert scale (1 = never, 2 = rarely, 3 = sometimes, 4 = often, and 5 = always). The score that can be collected from the scale can vary between 18 and 90. The score to be taken from all sub-dimensions is shown to have the features evaluated by the dimension that belongs to the student. It was stated that the scale supported the three-factor structure of the scale, Cronbach's alpha, two-half reliability coefficients of 0.88 and 0.85 were at an acceptable level for the scale, it had good construct validity, and the reliability and validity of the scale were said to be satisfactory for the Turkish sample (Aydin and Sütcü, 2007).

#### Rosenberg Self-Esteem Scale

Adapted by Çuhadaroglu, the first 10 items measure the self-esteem dimension and consist of 63 items and there are also 12 tests. Self-esteem test was used in the study. It is evaluated with options such as very right, right, wrong, very wrong as upper (0–1 points), low (5–6 points), and medium (2–4 points). A low score indicates high self-esteem, and a high score indicates low self-esteem. The reliability coefficient was found as 0.75 and the validity coefficient as 0.71 (Çeçen, 2008).

#### Personal information form

The personal information form developed for the students by the researcher included students' gender, class branch, number of siblings, marital status of parents, age, mother's work, father's work, and demographic variables.

### Data collection process

Permission was obtained from the Ministry of National Education on 13 April 2021 to collect the data from all regional secondary schools, after the approved ethics committee Exemption Form was obtained from the ethics committee of Cyprus International University. Within a week, all school principals were interviewed with permission from the ministry. In the interviews, the principals were informed about the purpose of the research and the data collection tools that would be used. After the approval of the principals, the students were made to complete online scales under the control of the teachers assigned by those principals. The students were informed by the researcher about the following.

Students will voluntarily participate in the study between 25 March 2022 and 31 March 2022 at their convenient time. They have the right to withdraw from the study at any time, and if they agree to participate and complete the study, the information they provide during the study will be used only for this study, the information will be kept confidential, and identity information will not be requested. The purpose of the study will be used, the answers they will give alone will be known, if they have any questions, and the name of the researcher and phone numbers are already stated.

### Analysis of data

IBM Statistical Package for Social Sciences (SPSS) 25.0 IBM AMOS and 21.0 software were used for statistical analysis of the study data.

The distribution of the demographic characteristics of the adolescents was determined by frequency analysis, and the descriptive statistics were shown by looking at the scores they got from the results of Conflict Resolution Needs, Social Anxiety for Adolescents, Psychological Abuse, and Rosenberg Self-Esteem Scale tests.

Adolescents' Conflict Resolution Needs, Social Anxiety for Adolescents, Emotional Abuse, and Rosenberg Self-Esteem Scale scores on the normal distribution were examined using the Kolmogorov–Smirnov test, and it was determined that they did not show a normal distribution. Non-parametric hypothesis testing was used in the study. During the comparison stage, Mann–Whitney *U* test was used if the independent variable consisted of 2 groups, and the Kruskal–Wallis H test was used if it was more than 2. The correlations among the students' Conflict Resolution Needs, Social Anxiety for Adolescents, Emotional Abuse, and scores obtained from the Rosenberg Self-Esteem Scale were examined with the Spearman's test. Structural equation modeling was used for descriptive analysis and model testing. Students' Conflict Resolution Needs, Social Anxiety for Adolescents, and Emotional Abuse Scale scores were analyzed with regression analysis and structural equation modeling to predict Rosenberg Self-Esteem Scale scores.

### Results

#### Findings related to sub-problems

The first sub-problem of the study: “Is there a significant difference between the scores of the students' conflict resolution needs scale, social anxiety scale for adolescents, Emotional Abuse Scale, Rosenberg Self-Esteem scale?” The data obtained within the scope of the question are given in Table 2.

**Table 2:** Conflict resolution needs of students, social anxiety for adolescents, emotional abuse, and Rosenberg Self-Esteem Scores from the scale.

	**N**	**χ**	**Sd**	**Min**	**Max**
Conflict Resolution Needs Scale	530	34.85	12.40	19	95
Fear of negative evaluation	530	16.49	7.76	7	35
General social situations fear, restlessness	530	10.65	4.86	5	25
New social situations fear, restlessness	530	16.96	5.77	6	30
Social Anxiety Scale for Adolescents	530	44.11	16.49	18	89
Intimidation/humiliation	530	15.53	2.79	9	27
Emotional responsiveness	530	9.13	1.49	6	17
Delinquency	530	9.69	1.96	6	17
Rejection/isolation	530	11.18	1.60	6	16
Emotional Abuse Scale	530	45.52	5.81	27	74
Rosenberg Self-Esteem	530	1.40	1.00	0	5,17

When the data from Table 2 are examined, the average score obtained from the Conflict Resolution Needs Scale of the students in the research is seen as 34.85 ± 12.40.

The average score of the students in the Social Anxiety Scale for Adolescents is 44.11 ± 16.49, the average score of the Fear of Negative Evaluation sub-dimension of the scale is 16.49 ± 7.76, the average score of the Fear in General Social Situations and Feeling Uneasy sub-dimension is 10.65 ± 4.86, and the average score of the sub-dimension Fear and Unrest in the New Social Situation was 16.96 ± 5.77. The average score of the adolescents in the study from the Emotional Abuse Scale was 45.52 ± 5.81, the average of the scores they got from the Mobbing/Humiliation sub-dimension of the scale was 15.53 ± 2.79, and the average score of the Emotional Response sub-dimension was 9.13 ± 1.49. The average score of the Decriminalization sub-dimension is 9.69 ± 1.96 and the average score of the Denial/Isolation sub-dimension is 11.18 ± 1.60.

The average score of the students from the Rosenberg Self-Esteem scale is 1.40 ± 1.00.

The correlations between the scores of the individuals in the study from the Conflict Resolution Needs, Social Anxiety for Adolescents, Emotional Abuse, and Rosenberg Self-Esteem Scales are given in Table 3.

**Table 3:** Relationships between conflict resolution needs of students, social anxiety for adolescents, emotional abuse, Rosenberg Self-Esteem Scores from the scale.

		**1**	**2**	**3**	**4**	**5**	**6**	**7**	**8**	**9**	**10**	**11**
**Conflict Resolution Needs Scale**	r	1	0.465	0.405	0.286	0.430	0.341	0.246	0.345	−0.107	0.301	0.417
	p	.	0.000^*^	0.000^*^	0.000^*^	0.000^*^	0.000^*^	0.000^*^	0.000^*^	0.013^*^	0.000^*^	0.000^*^
Negative fear of evaluation	r		1	0.704	0.669	0.912	0.255	0.258	0.296	−0.073	0.241	0.460
	p		.	0.000^*^	0.000^*^	0.000^*^	0.000^*^	0.000^*^	0.000^*^	0.095	0.000^*^	0.000^*^
In general social situations fear, restlessness	r			1	0.700	0.864	0.249	0.130	0.210	−0.088	0.170	0.450
	p			.	0.000^*^	0.000^*^	0.000^*^	0.003^*^	0.000^*^	0.043^*^	0.000^*^	0.000^*^
In new social situations fear and restlessness	r				1	0.878	0.203	0.127	0.221	0.014	0.179	0.302
	p				.	0.000^*^	0.000^*^	0.003^*^	0.000^*^	0.741	0.000^*^	0.000^*^
**For Adolescents Social Anxiety Scale**	r					1	0.257	0.197	0.278	−0.059	0.221	0.450
	p					.	0.000^*^	0.000^*^	0.000^*^	0.175	0.000^*^	0.000^*^
Intimidation/ humiliation	r						1	0.372	0.359	0.094	0.767	0.180
	p						.	0.000^*^	0.000^*^	0.030^*^	0.000^*^	0.000^*^
Emotional Reaction	r							1	0.442	0.187	0.626	0.220
	p							.	0.000^*^	0.000^*^	0.000^*^	0.000^*^
Delinquency	r								1	0.082	0.650	0.267
	p								.	0.059	0.000^*^	0.000^*^
Rejection/isolation	r									1	0.406	−0.189
	p									.	0.000^*^	0.000^*^
**Emotional Abuse Scale**	r										1	0.172
	p										.	0.000^*^
**Rosenberg Self-respect**	r											1
	p											.

* p < 0,05.

According to the data obtained from Table 3, positive and meaningful correlations were found between the students' scores from the Conflict Resolution Needs Scale and the scores obtained from the Social Anxiety Scale for Adolescents in general, which are the sub-dimensions of the scale, Fear of Negative Evaluation, Fear in General Social Situation, Anxiety in a New Social Situation, Fear in a New Social Situation, and Anxiety in a New Social Situation (*p* < 0.05). As the scores of the Adolescents on the Conflict Resolution Needs Scale increase, the scores on the Social Anxiety Scale for Adolescents and all sub-dimensions of the scale also increase.

There are meaningful and positive correlations between the scores of the individuals in the study from the Conflict Resolution Needs Scale and the scores they got from the Emotional Abuse Scale in general; accordingly, as the scores that individuals get from the Conflict Resolution Needs Scale increase, the scores they get from the Emotional Abuse Scale in general and the sub-dimensions of Mobbing/Humiliation, Emotional Response, and Guilt also increase. There are meaningful and negative correlations between the scores of individuals from the Conflict Resolution Needs Scale and the scores they get from the Rejection/Isolation sub-dimension of the Emotional Abuse Scale (*p* < 0.05). As the scores of the adolescents on the Conflict Resolution Needs Scale increase, their Rejection/Isolation scores decrease.

Meaningful and positive correlations were found between the scores the students got from the Conflict Resolution Needs Scale and the scores they got from the Rosenberg Self-Esteem Scale (*p* < 0.05). Accordingly, as the Conflict Resolution Needs Scale scores of the individuals increase, the Rosenberg Self-Esteem Scale scores also increase.

It was determined that there were meaningful and positive correlations between the scores of the students in the Social Anxiety Scale for Adolescents and all sub-dimensions of the scale and the scores they got from the Emotional Abuse Scale in general and the sub-dimensions of Mobbing/Humiliation, Emotional Response, and Guilt (*p* < 0.05).

Accordingly, as the students' general Social Anxiety Scale for Adolescents and Fear of Negative Evaluation, Fear and Unrest in General Social Situations, and Fear and Unrest in New Social Situations increase, the Emotional Abuse Scale and Mobbing/Humiliation, Emotional Response, and Guilt-Orientation scores increase as well. In addition, statistically meaningful and negative correlations were found between the scores of the students in the sub-dimension of Fear and Restlessness in General Social Situations and the scores they got from the Rejection/Isolation sub-dimension of the Emotional Abuse Scale (*p* < 0.05); It was observed that as the scores of Fear and Restlessness in General Social Situations increased, the Rejection/Isolation scores decreased.

It was determined that there were significant and positive correlations between the scores of the individuals in the study from the Social Anxiety Scale for Adolescents in general and from all sub-dimensions of the scale and the scores they got from the Rosenberg Self-Esteem Scale (*p* < 0.05). Accordingly, as the students' scores on the Social Anxiety Scale for Adolescents in general and on all sub-dimensions of the scale increase, their Rosenberg Self-Esteem scores also increase.

Statistically significant and positive correlations were found between the scores the students got from the Emotional Abuse Scale in general and the sub-dimensions of Mobbing/Humiliation, Emotional Response, and Guilt and the scores they got from the Rosenberg Self-Esteem Scale (*p* < 0.05). Accordingly, as the overall Emotional Abuse Scale and Mobbing/Humiliation, Emotional Response, and Criminalization scores of the individuals in the study increase, their Rosenberg Self-Esteem scores also increase. In addition, significant and negative correlations were observed between the scores of the students in the Rejection/Isolation sub-dimension and the Rosenberg Self-Esteem Scale scores (*p* < 0.05); it was determined that as the rejection/isolation scores increased, the Rosenberg Self-Esteem scores decreased.

When the data obtained within the scope of the question: “Is there a meaningful difference in the prediction level of the scores of the students' Conflict Resolution Needs Scale scores, which are the second sub-problems of the research, from the Rosenberg Self-Esteem Scale scores?” are examined, the results of the linear regression analysis are given in Table 4.

**Table 4:** Prediction of the students' conflict resolution needs scale scores from the Rosenberg Self-Esteem Scale Scores.

	**Not standard coefficients**		**Standardized coefficients**	**T**	**p**	**F**	**R^2^**
	**B**	**S.H**.	**Beta**			**P**	**AdjR^2^**
(Stable)	0.05	0.11		0.462	0.644	157.923	0.230
**Conflict Resolution Needs Scale**	0.04	0.00	0.48	12.567	0.000*	0.000*	0.229

Looking at Table 4, it is seen that the Conflict Resolution Needs Scale scores of the adolescents meaningfully and positively predicted the scores they got from the Rosenberg Self-Esteem Scale (β = 0.48; *p* < 0.05).

The third sub-problem of the study is, “Is there a significant difference in the predictive level of the scores of the students on the Social Anxiety Scale for Adolescents and the scores they got from the Rosenberg Self-Esteem Scale?” The result of the linear regression analysis, in which the prediction status was examined within the scope of the question, is given in Table 5.

**Table 5:** Prediction of the social anxiety scale for adolescents scores of the students and the scores they got from the Rosenberg Self-Esteem Scale.

	**Not being standardized coefficients**		**Standardized coefficients**	**T**	**p**	**F**	**R^2^**
	**B**	**S.H**.	**Beta**			**P**	**AdjR^2^**
(Stable)	0.13	0.11		1.190	0.235	154.068	0.226
**Social Anxiety Scale for Adolescents**	0.03	0.00	0.48	12.412	0.000^*^	0.000^*^	0.224

* p < 0,05.

Looking at Table 5, it is seen that the Social Anxiety Scale for Adolescents scores of the students included in the study predicted the scores they got from the Rosenberg Self-Esteem Scale in a statistically meaningful and positive direction (β = 0.48; *p* < 0.05).

The fourth sub-problem of the study is, “Is there a meaningful difference in the predictive level of the students' Emotional Abuse Scale scores and the Rosenberg Self-Esteem Scale scores?” The result of the linear regression analysis, in which the prediction status was examined within the scope of the question, is given in Table 6.

**Table 6:** Prediction of the emotional abuse scale scores of the students and the scores they got from the Rosenberg Self-Esteem Scale.

	**Coefficients of being standard**		**Standardized coefficients**	**T**	**p**	**F**	**R^2^**
	**B**	**S.H**.	**Beta**			**P**	**AdjR^2^**
(Stable)	−0.60	0.33		−1.803	0.072	36.841	0.065
**Adolescents Anxiety Scale for Adolescents**	0.04	0.01	0.26	6.070	0.000^*^	0.000^*^	0.063

* p < 0,05.

When Table 6 is examined, it was seen that the Emotional Abuse Scale scores of the students predicted the scores they got from the Rosenberg Self-Esteem Scale in a statistically meaningful and positive way (β = 0.26; *p* < 0.05).

As can be seen above, it has been determined that the Conflict Resolution Needs Scale, the Social Anxiety Scale for Adolescents, and the Emotional Abuse Scale scores alone predict the scores the students get from the Rosenberg Self-Esteem Scale. Accordingly, it was deemed appropriate to examine the mediating role of students' Conflict Resolution Needs Scale scores between Social Anxiety and Emotional Abuse Scale for Adolescents scores and Rosenberg Self-Esteem Scale scores, and thus, structural equation modeling was used in this regard.

For the fifth sub-problem of the study is, “Do students' Conflict Resolution Needs Scale scores have a mediating role between Adolescent Social Anxiety and Psychological Abuse Scale scores and Rosenberg Self-Esteem Scale scores?” the data obtained within the scope of the question are given in Table 7.

**Table 7:** Goodness-of-fit indexes of the model regarding the mediator role of students' Conflict Resolution Needs Scale Scores between social anxiety and Psychological Abuse Scale Scores for adolescents and Rosenberg Self-Esteem Scale Scores.

**Goodness-of-fit indexes**	**Calculate value**	**Compliance**
χ^2^/sd (chi square / degrees of freedom)	3.814	Acceptable
Root Mean Square Errors Of Approximate (RMSEA)	0.073	Acceptable
Normalized Compliance Index (NFI)	0.963	Excellent
Comparative Fit Index (CFI)	0.972	Excellent
Goodness-of-Fit Index (GFI)	0.969	Excellent
Flat. Goodness-of-Fit Index (AGFI)	0.931	Acceptable

Considering the goodness-of-fit indexes of the model regarding the mediator role of the students' Conflict Resolution Needs Scale scores given in Table 7, the χ^2^/sd (chi-square / degree of freedom) of the established model was determined to be 3,814, and it was seen that the model was in an acceptable fit. The value of Kline (2005) χ^2^/sd being below 3 indicates a perfect fit, being between 3 and 5 an acceptable fit.

Brown (2006) stated that RMSEA value between 0.00 and 0.05 indicates perfect fit and between 0.05 and 0.08 indicates acceptable fit. Root Mean Square Errors of Approximate (RMSEA) value was determined to be 0.073, and in this case, it was determined that the model was within acceptable fit limits.

The normed fit index (NFI) was determined to be 0.963, the comparative fit index (CFI) was determined as 0.972, and the goodness-of-fit index (GFI) value was 0.969. Tabachnick and Fidell (2001) stated that the acceptable limit for these values was between 0.90 and 0.95, and the perfect fit was 0.95–1.0. Accordingly, the model has a perfect fit in terms of NFI, CFI, and GFI.

As can be seen above, it was determined that the goodness-of-fit indexes of the model created regarding the mediating role of the students' Conflict Resolution Needs Scale scores between the Social Anxiety and Psychological Abuse Scale for Adolescents scores and the Rosenberg Self-Esteem Scale scores were found to be sufficient.

**Figure 1:**
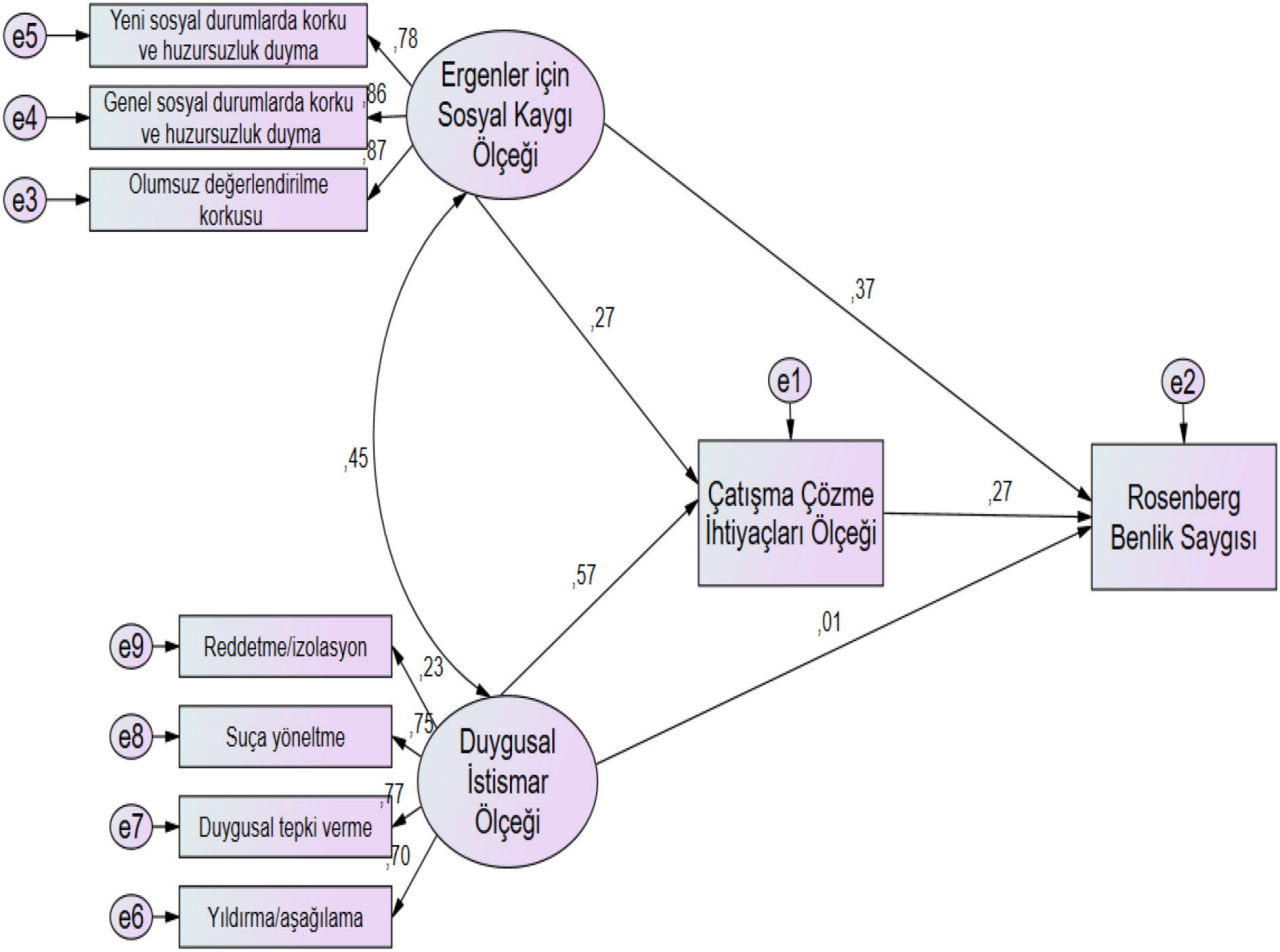
The mediating role of students' Conflict Resolution Needs Scale scores between Social Anxiety and Emotional Abuse Scale for Adolescents scores and Rosenberg Self-Esteem Scale scores.

When the model shown in Figure 1 is examined, it is observed that the Social Anxiety Scale for Adolescents scores of the students participating in the study predicted the Conflict Resolution Needs Scale scores in a statistically meaningful and positive direction (β = 0.27; *p* < 0.05). It was observed that the Conflict Resolution Needs Scale scores of the students participating in the study predicted the scores they got from the Rosenberg Self-Esteem Scale at a statistically meaningful level (β = 0.27; *p* < 0.05). Finally, it was determined that the Social Anxiety Scale for Adolescents scores also predicted the scores they got from the Rosenberg Self-Esteem Scale at a statistically meaningful level (β = 0.37; *p* < 0.05). As previously determined, students' scores on the Adolescent Social Anxiety Scale alone predict their Rosenberg Self-Esteem scores. When the Conflict Resolution Needs Scale was included in the model, it was observed that the mentioned prediction situation continued. Accordingly, it has been determined that Conflict Resolution Needs do not have a mediating role between Social Anxiety and Self-Esteem. It was observed that students' Emotional Abuse Scale scores positively predicted their Conflict Resolution Needs Scale scores at a statistically meaningful level (β = 0.57; *p* < 0.05). It was determined that the Conflict Resolution Needs Scale scores of the students participating in the study predicted the scores they got from the Rosenberg Self-Esteem Scale at a statistically meaningful level (β = 0.27; *p* < 0.05). Emotional Abuse Scale scores were also found to not statistically meaningfully predict the scores the students got from the Rosenberg Self-Esteem Scale (β = 0.01; *p* > 0.05). It was determined that the scores the students got from Psychological Abuse Scale alone predicted their Rosenberg Self-Esteem scores, and when the Conflict Resolution Needs Scale was included in the model, the mentioned prediction situation disappeared. Accordingly, it has been determined that Conflict Resolution Needs have a mediating role between Emotional Abuse and Self-Esteem.

## Results

There are significant and positive correlations between the scores individuals get from the Conflict Resolution Needs Scale and the scores they get from Emotional Abuse Scale in general and the sub-dimensions of Mobbing/Humiliation, Emotional Response, and Guilt (*p* < 0.05). Accordingly, as the scores that individuals get from the Conflict Resolution Needs Scale increase, the scores they get from the Emotional Abuse Scale in general and from the sub-dimensions of Mobbing/Humiliation, Emotional Response, and Criminalization also increase.

Meaningful and positive correlations were found between the scores the students got from the Conflict Resolution Needs Scale and the scores they got from the Rosenberg Self-Esteem Scale (*p* < 0.05). Accordingly, as the Conflict Resolution Needs Scale scores of the individuals increase, the Rosenberg Self-Esteem Scale scores also increase.

It was determined that there were significant and positive correlations between the scores of the students in the Social Anxiety Scale for Adolescents and all sub-dimensions of the scale and the scores they got from the Emotional Abuse Scale in general and the sub-dimensions of Mobbing/Humiliation, Emotional Response, and Guilt (*p* < 0.05). Accordingly, as the students' general Social Anxiety Scale for Adolescents and Fear of Negative Evaluation, Fear and Unrest In General Social Situations, Fear and Unrest in New Social Situations increase, the Emotional Abuse Scale and Mobbing/Humiliation, Emotional Response, and Guilt-Orientation scores increase as well.

It was determined that there were significant and positive correlations between the scores the individuals got from the study of Social Anxiety Scale for Adolescents in general and from all sub-dimensions of the scale and the scores they got from the Rosenberg Self-Esteem Scale (*p* < 0.05). Accordingly, as the students' scores on the Social Anxiety Scale for Adolescents in general and on all sub-dimensions of the scale increase, their Rosenberg Self-Esteem scores also increase.

Statistically significant and positive correlations were found between the scores the students got from the Emotional Abuse Scale in general and the sub-dimensions of Mobbing/Humiliation, Emotional Response, and Guilt and the scores they got from the Rosenberg Self-Esteem Scale (*p* < 0.05). Accordingly, as the overall Emotional Abuse Scale and Mobbing/Humiliation, Emotional Response, and Criminalization scores of the individuals in the study increase, the Rosenberg Self-Esteem scores also increase.

## Discussion

In order for middle school students who are in adolescence to get through this period in a healthy way, the most important point for the adolescent is to get support from their mother, father, school, teacher, and environment and to receive the love, knowledge, and trust they need (Durmuşoglu and Dogru, 2006). In the study of the Investigation of the Effects of Childhood Abusive Experiences on the Individual in Close Relationships in Adolescence, it was stated that close relationships contribute to the development of parents, teachers, friends, and environment self-esteem, and that adolescents need this close relationship to satisfy their needs and solve the problems they experience in every period, which supports this study.

It has been observed that adolescents who need conflict resolution are afraid of negative evaluation, general social situations, new social situations and they increasingly feel uneasy, and their social anxiety increases, which leads them to react emotionally and turn to crime. In other studies, it has been shown that problem-solving skills increase as social support from family, teachers, and friends increases. As the social support he/she receives from her/his friends increases, her/his social development will increase and she/he will be able to establish better relationships. There is a necessity of dealing with conflict resolution bullying micro-, mezzo-, macro-dimension, family, student, and school with a holistic approach; when the students' self-perceptions are examined according to their relationship with their environment and friends, it has been observed that students who have very good relations with their environment and friends have high self-perceptions (Sezer, 2010; Öztürk, 2019; Karapinar, 2021).

When the literature is examined, it has been observed that as the perceived emotional abuse from the parents increases, the social, academic, and emotional self-efficacy levels of the students decrease. Adolescents who do not perceive emotional abuse from their parents are more socially and personally compatible; the adolescent exposed to emotional abuse tends to become closer to external control and be more anxious, whereas the adolescent who is not emotionally abused is closer to internal control and less anxious; there is a significant relationship between emotional abuse and self-esteem of students' conflict resolution skills, problem-solving, and social support; It was found that there is a meaningful positive relationship among teacher, family, and friends (Durmuşoglu and Dogru, 2006; Yalçin, 2007; Öztep, 2010; Koçmarlar and Akbag, 2018; Mert et al., 2019; Karapinar, 2021).

In the study conducted by Aslan and Koç (2018) with 783 adolescents aged between 14 and 18 years at 2018, it was observed that there is a full correlation between the problems of internalization and externalization, in which they use the avoidance strategy rather than the active strategy, which affects the mental health of the adolescent that experiences them. It has been emphasized that adolescents who have been abused at a high level use maladaptive coping strategies and this affects their mental and behavioral movements. He supported the study by suggesting that the parent–child relationship is important in the development of coping strategies, he will learn how to cope with stressful events at a young age; when problems occur, serious negative behaviors can result in adolescents, where prevention and intervention services can be offered to young people along with parent help starting from childhood.

By looking at the findings of the research and literature review, it can be suggested that students who get support from their schools, families, friends, and social circles in cooperation with their skills and activities can overcome the problems of negative evaluation, uneasiness, anxiety, and fear in social situations.

High support from parents: Positive esteem is associated with consistent intelligence and self-efficacy, and adolescents with a secure self-esteem will be less likely to give up when faced with problems (Frank et al., 2010). In a study conducted with 1,041 Chinese students between the ages of 11 and 15, it was seen that child neglect and emotional abuse were effective on students' coping styles and emotional intelligence (Sun et al., 2019). In the study examining parent–conflict relationships, it was found that students thought that most of the conflicts were initiated by their parents or siblings and that these conflicts happened every day. He stated in their suggestions that they did not use a systematic method for conflict resolution and that students could resolve conflicts with structured guidance (Riesch et al., 2003). It was observed that students with better student–peer relationships had higher self-esteem, whereas students with low self-esteem had worse student–peer relationships (Wang et al., 2017). In the study conducted to examine the mediating role of self-esteem in students' emotional abuse and behavioral problems, it was found that students who experienced emotional abuse had low self-esteem and behavioral problems (Arslan, 2016). The results of the research contribute to science.

## Data availability statement

The original contributions presented in the study are included in the article/supplementary material, further inquiries can be directed to the corresponding author.

## Author contributions

While writing the article, all findings and researches were made and analyzed and the results were interpreted by TE. The results of the study were reviewed and approved by the thesis supervisor YK. All authors contributed to the article and approved the submitted version.

## Conflict of interest

The authors declare that the research was conducted in the absence of any commercial or financial relationships that could be construed as a potential conflict of interest.

## Publisher's note

All claims expressed in this article are solely those of the authors and do not necessarily represent those of their affiliated organizations, or those of the publisher, the editors and the reviewers. Any product that may be evaluated in this article, or claim that may be made by its manufacturer, is not guaranteed or endorsed by the publisher.
